# A Comparison of Multidrug-Resistant Tuberculosis Treatment Commencement Times in MDRTBPlus Line Probe Assay and Xpert® MTB/RIF-Based Algorithms in a Routine Operational Setting in Cape Town

**DOI:** 10.1371/journal.pone.0103328

**Published:** 2014-07-31

**Authors:** Pren Naidoo, Elizabeth du Toit, Rory Dunbar, Carl Lombard, Judy Caldwell, Anne Detjen, S. Bertel Squire, Donald A. Enarson, Nulda Beyers

**Affiliations:** 1 Desmond Tutu TB Centre, Department of Paediatrics and Child Health, Faculty of Medicine and Health Sciences, Stellenbosch University, Cape Town, South Africa; 2 Medical Research Council, Cape Town, South Africa; 3 Cape Town Health Directorate, Cape Town, South Africa; 4 The International Union Against TB and Lung Disease, Paris, France; 5 Liverpool School of Tropical Medicine, Liverpool, United Kingdom; University of Cape Town, South Africa

## Abstract

**Background:**

Xpert MTB/RIF was introduced as a screening test for all presumptive tuberculosis cases in primary health services in Cape Town, South Africa.

**Study Aim:**

To compare multidrug-resistant tuberculosis (MDR-TB) treatment commencement times in MDRTBPlus Line Probe Assay and Xpert MTB/RIF-based algorithms in a routine operational setting.

**Methods:**

The study was undertaken in 10 of 29 high tuberculosis burden primary health facilities, selected through stratified random sampling. An observational study was undertaken as facilities transitioned to the Xpert MTB/RIF-based algorithm. MDR-TB diagnostic data were collected from electronic laboratory records and treatment data from clinical records and registers. Kaplan Meier time-to-event analysis was used to compare treatment commencement time, laboratory turnaround time and action delay between algorithms. A facility-level paired analysis was done: the median time-to-event was estimated per facility in each algorithm and mean differences between algorithms compared using a paired t-test. Cox proportional hazards regression was used to assess the effect of patient-level variables on treatment commencement time. The difference between algorithms was compared using the hazard ratio.

**Results:**

The median treatment commencement time in the Xpert MTB/RIF-based algorithm was 17 days (95% CI 13 to 22 days), with a median laboratory turnaround time (to result available in the laboratory) of <1 day (95% CI<1 to 1 day). There was a decrease of 25 days (95% CI 17 to 32 days, p<0.001) in median MDR-TB treatment commencement time in the Xpert MTB/RIF-based algorithm. We found no significant effect on treatment commencement times for the patient-level variables assessed.

**Conclusion:**

MDR-TB treatment commencement time was significantly reduced in the Xpert MTB/RIF-based algorithm. Changes in the health system may have contributed. However, an unacceptable level of delay remains. Health system and patient factors contributing to delay need to be evaluated and addressed to optimise test benefits.

## Introduction

Improving multidrug-resistant tuberculosis (MDR-TB) control requires access to accurate and rapid diagnostics for drug susceptibility testing [1]–[3]. A rapid diagnosis has both patient and public health benefits: it enables early, appropriate treatment which can reduce morbidity and mortality for patients as well as transmission within communities. This is of particular importance in South Africa which has a high TB and MDR-TB burden with 349, 582 and 15,419 cases respectively reported in 2012 [4]. South Africa’s early adoption of new molecular diagnostic tests is one of the responses to the TB crisis: Hain-MDRTBPlus line probe assay (LPA) was introduced following the WHO Policy statement in 2008 [5] and Xpert MTB/RIF (Xpert) following the 2011 policy statement [6].

The efficacy of both tests has been well established [7]–[12] and confirmed by systematic reviews [13]–[15]. Policy recommendations for both diagnostics have been based largely on accuracy data from laboratory and demonstration studies. This has limitations, as test performance under operational conditions and evidence linking accuracy to patient important outcomes are not considered, making it difficult to translate “scientific progress into public health impact” [16].

Few studies have reported on the effect of molecular diagnostics on MDR-TB treatment delay [17]–[19]. Studies from South Africa that compared conventional drug susceptibility tests (DST) to LPA showed a reduction in median treatment commencement time from 72 days with conventional DST to 24 days with LPA in a demonstration study [17] and from 80 to 55 days in a rural TB hospital [18]. Although studies have reported a reduction in treatment delay with Xpert for drug-susceptible cases [12], [20], we are not aware of any publications that address its effect on MDR-TB treatment delay.

This study is part of a broader PROVE IT (**P**olicy **R**elevant **O**utcomes from **V**alidating **E**vidence on **I**mpac**T**) (http://treattb.org) evaluation undertaken in Cape Town, South Africa, to assess the impact of new molecular diagnostics on the diagnosis and treatment of tuberculosis. Guided by the Impact Assessment Framework [21], the magnitude and range of benefits for patients (from clinical presentation to treatment initiation), the magnitude and nature of inputs required and factors that influence policy change were evaluated.

### Study Aim

We compared MDR-TB treatment commencement times (TCT) in LPA and Xpert-based algorithms in a routine operational setting. Treatment non-initiation rates and the association between MDR-TB TCT and patient level variables such as age, sex, HIV-status, MDR-risk profile, MDR-TB diagnostic time-point and treatment initiation site were also assessed.

## Methods

This observational cohort study compared cases in a LPA-based algorithm to those in an Xpert-based algorithm, as facilities transitioned to the latter in 2011–2012.

### Setting

The study was undertaken in Cape Town, South Africa. The City had a high TB burden with 28,658 TB cases and 953 MDR-TB cases recorded in 2011 and a TB case notification rate of 752/100,000 population (Source: J. Caldwell, Routine TB Programme Data, Cape Town Health Directorate). Free TB diagnostic services were provided at 142 primary health care (PHC) facilities and treatment at 101 of these. A daily courier delivered all specimens to a central laboratory where tests were done and results recorded in an electronic laboratory database. Positive TB results were faxed to facilities on a daily basis and hard copies of all results returned by courier.

Patients diagnosed with MDR-TB received standardised treatment regimens. Historically, patients initiated MDR-TB treatment at a central TB-hospital. From 2005, doctors at the TB hospital reviewed case records and prescribed treatment but patients could initiate treatment at PHC facilities. In 2012, doctors at the PHC facilities offering TB treatment initiated standardised MDR-TB treatment without the need for prior review of the case at the TB-hospital.

### TB Algorithms

**Figure 1:**
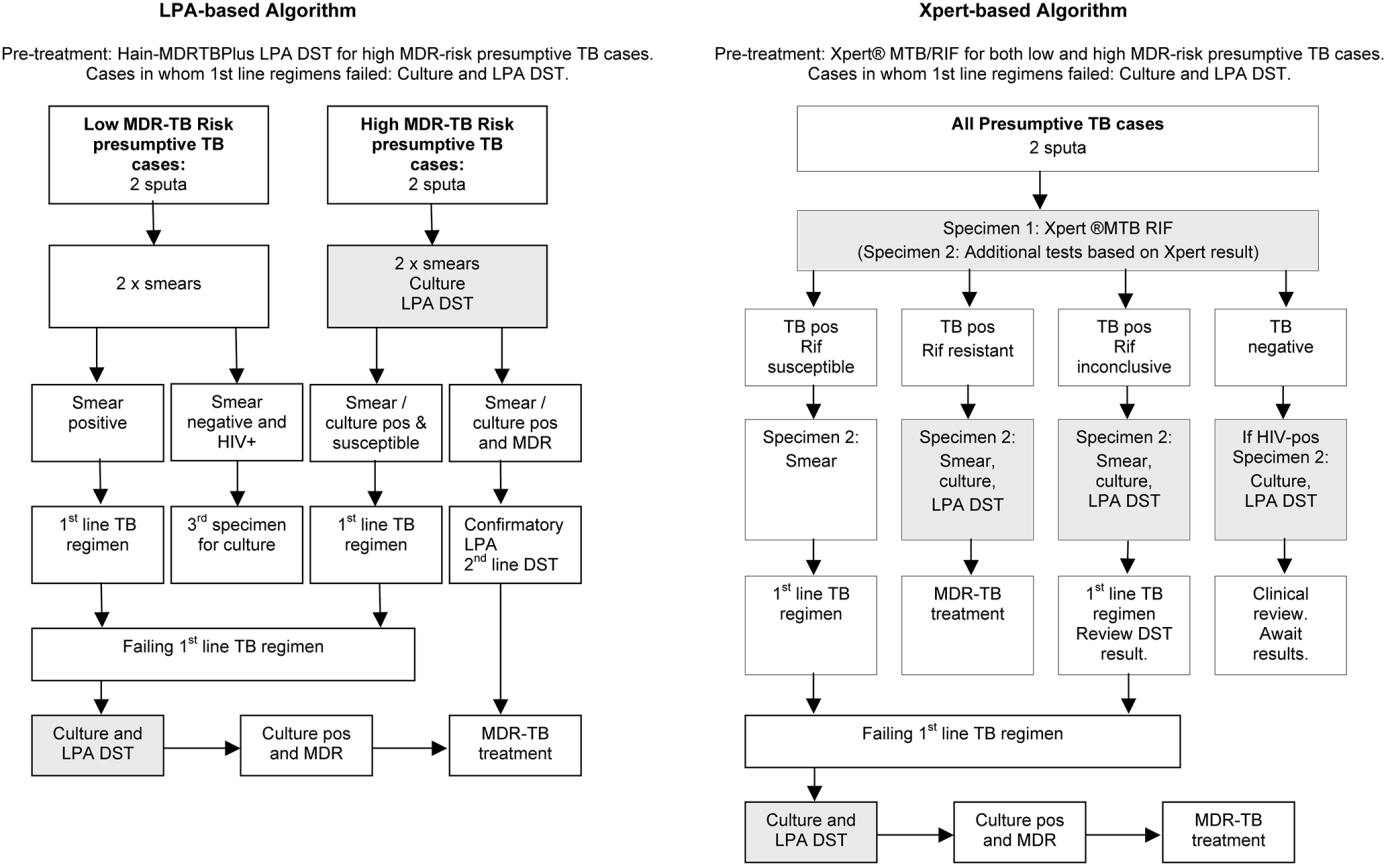
TB Testing in the LPA and Xpert-based Algorithms. The sequence of diagnostic tests in each algorithm and the action taken based on test results is shown. Shaded blocks indicate possible MDR-TB diagnostic points. Abbreviations: MDR-TB - multidrug-resistant tuberculosis; LPA - line probe assay; DST - drug susceptibility testing; HIV – human immunodeficiency virus; Rif – rifampicin; Pos – positi.

The health department introduced LPA as a replacement for conventional first-line DST in January 2008. LPA was initially performed on culture (BACTEC MGIT 960) isolates in *high MDR-risk* presumptive TB cases and later also directly on smear-positive sputa and referred to in this study as the LPA-based algorithm (Figure 1).

From 2011 to 2013 Xpert was sequentially introduced into the eight health sub-districts in Cape Town, replacing smear microscopy for *all* presumptive TB cases and referred to in this study as the Xpert-based algorithm (Figure 1).

In both algorithms, cases in whom 1^st^ line regimens failed (i.e. those with positive smears during the course of treatment and or clinical deterioration) were evaluated for MDR-TB though culture and LPA (Figure 1).

### Study Population

This study was undertaken in a routine operational setting in 10 high TB-burden government PHC facilities, selected from a total of 29 that met the criteria of a TB caseload of >350 in 2009. Two facilities were excluded due to competing research studies. The remaining facilities were ordered according to their smear-positive treatment success rates in 2009 and five were randomly selected from each group above and below the median.

**Figure 2:**
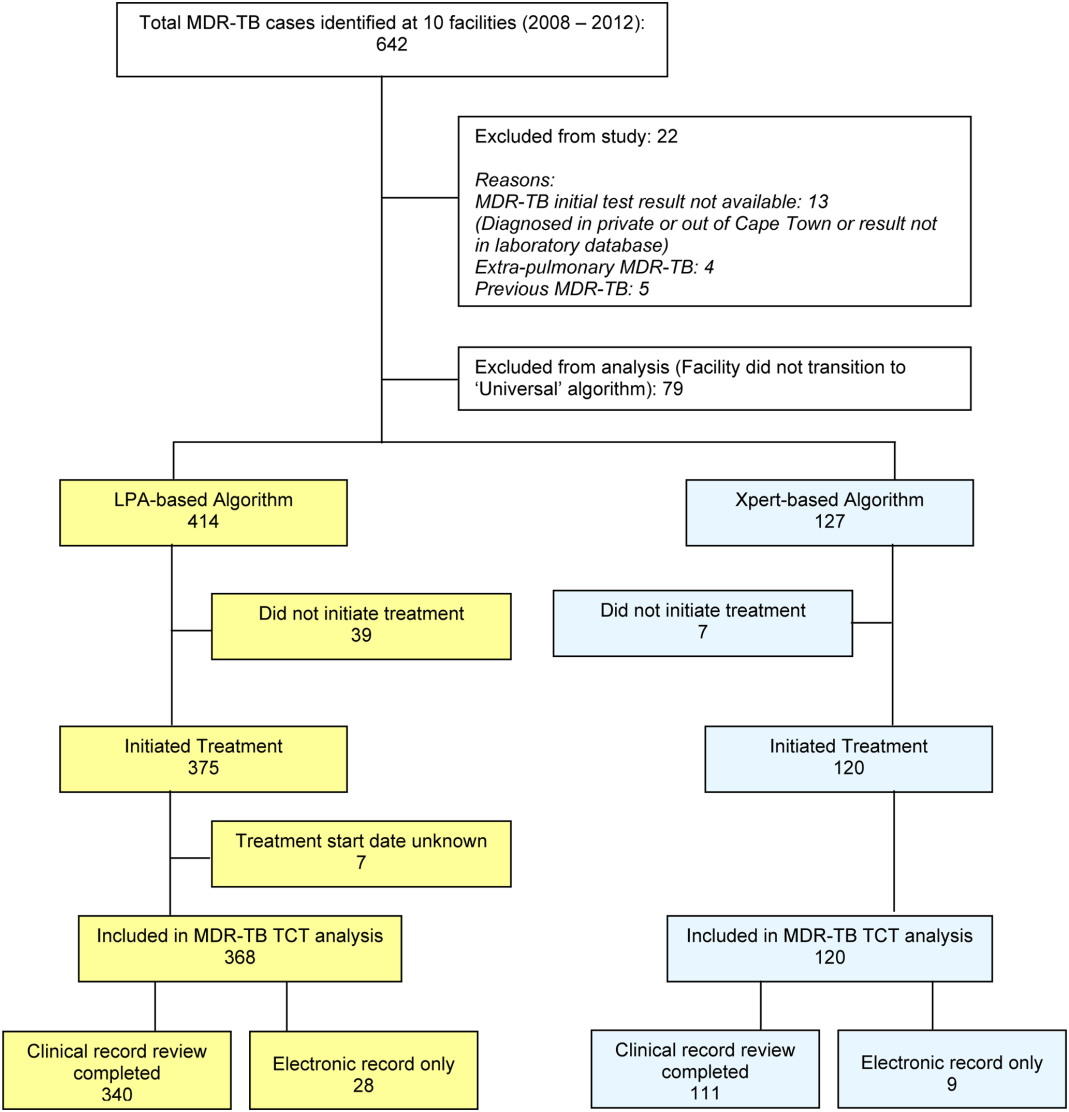
Study Population. MDR-TB cases identified from selected facilities and those included and excluded from the study and from the final analysis are shown. Abbreviations: MDR-TB – multidrug-resistant tuberculosis; DST- drug susceptibility test; TCT - treatment commencement time.

All individuals with sputum samples taken at these facilities between January 2008 and December 2012 and with a laboratory diagnosis of pulmonary MDR-TB were included in the study (Figure 2). Cases diagnosed at other public health facilities in Cape Town that had received treatment at the selected facilities were also included. Cases with previous MDR-TB treatment or without results from the national health laboratory were excluded. Only cases from the 9 facilities that transitioned to the Xpert-based algorithm in the study period were included in the analysis.

### Data Sources, Collection and Management

Patients diagnosed with MDR-TB in selected facilities were identified from the electronic laboratory database using the facility name and location code; those diagnosed elsewhere, but on treatment at the selected facilities, were identified through facility DR-TB paper registers and patient clinical records. Professional nurses undertook clinical record reviews of all cases that met the inclusion criteria and recorded demographic, laboratory and clinical data on case report forms. Data were quality checked, dual entered into a Microsoft SQL database and corrected. Where clinical records could not be found, treatment data were extracted from electronic in-patient records at the TB hospital and from sub-district electronic DR-TB registers. Data from these sources were linked to electronic laboratory data, which provided information on the specimen tested, test dates, type of test and results.

Study data will be made available to other researchers on request, with permission from the relevant authorities.

### Definitions

In both algorithms, a specimen with rifampicin and isoniazid resistance was defined as MDR-TB; in the Xpert-based algorithm, rifampicin-resistance on Xpert was considered a proxy indicator of MDR-TB.

The primary outcome measure of *MDR-TB TCT* was calculated from first MDR-TB diagnostic sputum collection date to MDR-TB treatment commencement date and comprised two intermediary times:

1. *Laboratory turnaround time* was calculated from date of sputum collection to date result was available in the laboratory.
2. *‘Action’ delay* was calculated from date result was available in the laboratory to treatment commencement date.

*Non-initiation* of MDR-TB treatment was defined as no record of treatment initiation in facility records, the electronic DR-TB register or the in-patient hospital database within 6 months of the MDR-TB test sputum being collected.

The *MDR-TB diagnostic time-point* was defined as either *pre-treatment,* for a presumptive TB case being concurrently evaluated for TB and drug resistance, or as *treatment,* for a case on a 1st-line TB regimen being evaluated for drug resistance.

*Low MDR-TB risk* was defined as ≤four weeks previous TB treatment and *high MDR-TB risk* as >four weeks previous TB treatment, from congregate settings or with a known MDR-TB contact, based on clinical records.

### Statistical Analysis

Demographic characteristics were analyzed using the t-test for normally distributed continuous outcomes and chi-square for categorical outcomes. Kaplan Meier time-to-event analysis was used to compare treatment commencement time, laboratory turnaround time and action delay between algorithms. Kaplan Meier survival distribution was used to estimate median time-to-event, defined as the length of time corresponding to the probability of 0.5 [22]. A facility-level paired analysis was used to generate summary statistics: the median time-to-event was estimated per facility and mean differences between diagnostic algorithms compared using a paired t-test.

Cox proportional hazards regression using the Breslow method for ties with a facility-level stratification was used to assess the effect of patient-level variables such as age, gender, HIV-status, MDR-TB risk profile and treatment initiation site on MDR-TB TCT. We adjusted for these variables and used the hazard ratio to compare the overall difference in MDR-TB TCT between diagnostic algorithms. Analyses were undertaken using STATA 12 (StataCorp).

### Ethics Statement

The City Health Directorate, Western Cape Health Department and National Health Laboratory Service granted permission to use the routine health data. The Health Research Ethics Committee at Stellenbosch University (IRB0005239) (N10/09/308) and Ethics Advisory Group at The International Union Against Tuberculosis and Lung Disease (59/10) approved the study. A waiver of informed consent was granted for the use of routine data.

## Results

Of the 642 MDR-TB cases identified, 541 met the criteria for inclusion in the analysis (Figure 2). Amongst the 414 cases in the LPA-based and 127 in the Xpert-based algorithm, there were no significant differences in sex, age, HIV-status or MDR-TB risk profile (Table 1). In the LPA-based algorithm, 68% were diagnosed at the pre-treatment MDR-TB diagnostic time-point compared to 83% in the Xpert-based algorithm (p = 0.002).

**Table 1:** Comparison of Baseline Characteristics of MDR-TB Cases by Algorithm.

		Total Cohort			Did Not Initiate Treatment			Initiated Treatment		
		LPA-basedAlgorithm	Xpert-basedAlgorithm	*p-value*	LPA-basedAlgorithm	Xpert-basedAlgorithm	*p-value*	LPA-basedAlgorithm	Xpert-basedAlgorithm	*p-value*
Total cases		414	127		39 (9%)	7 (6%)	*0.167*	375 (91%)	120 (94%)	
Sex	Female n (%)	184 (44%)	53 (42%)	*0.590*	15 (38%)	5 (71%)	*0.213*	169 (45%)	48 (40%)	*0.330*
	Male n (%)	230 (56%)	74 (58%)		24 (62%)	2 (29%)		206 (55%)	72 (60%)	
Age	Mean (Years)	35	35	*0.483*	35	35	*0.504*	35	35	*0.478*
	SD (Years)	11	11		12	9		11	11	
	Range (Years)	8–81	12–68		19–81	25–53		8–71	12–68	
HIV-status	HIV-positive n (%)	216 (59%)	71 (60%)	*0.828*	17 (85%)	3 (75%)	*0.624*	199 (57%)	68 (59%)	*0.691*
	HIV-negative n (%)	153 (41%)	48 (40%)		3 (15%)	1 (25%)		150 (43%)	47 (41%)	
MDR-TBrisk profile	Low-risk n (%)	155 (38%)	59 (46%)	*0.077*	16 (44%)	3 (43%)	*1.000*	138 (37%)	56 (47	*0.059*
	High-risk n (%)	255 (62%)	68 (54%)		20 (56%)	4 (57%)		235 (63%)	64 (53%)	
MDR-TBdiagnostictime-point	Pre-treatment n (%)	253 (68%)	101 (83%)	*0.002*	19 (79%)	5 (71%)	*0.667*	234 (67%)	96 (83%)	*0.001*
	Treatment n (%)	118 (32%)	21 (17%)		5 (21%)	2 (29%)		113 (33%)	19 (17%)	
Treatmentinitiation site	TB Hospital n (%)							43 (12%)	2 (2%)	*<0.001*
	PHC Facility n (%)							313 (88%)	114 (98%)	

Percentages shown were calculated based on recorded data only. Missing data is not reflected but can be calculated based on totals in the cohort and the recorded data shown. Abbreviations: HIV – human immunodeficiency virus; MDR-TB – multi-drug resistant tuberculosis; PHC – primary health care.

Non-initiation rates did not differ between the LPA (9%) and Xpert-based algorithm (6%) (risk ratio 1.7, p = 0.167). Comparative data for those who did and did not initiate treatment found that only HIV contributed significantly to non-initiation in the LPA-based algorithm (85% HIV-positive in the non-initiation group compared to 57% for those on treatment, p = 0.014).

Amongst cases on MDR-TB treatment (Table 1), there were no significant differences in sex, age, HIV-status or MDR-TB risk profile between the algorithms. More patients initiated treatment at PHC-level in the Xpert (98%) than in the LPA-based algorithm (88%) (p<0.001).

**Figure 3:**
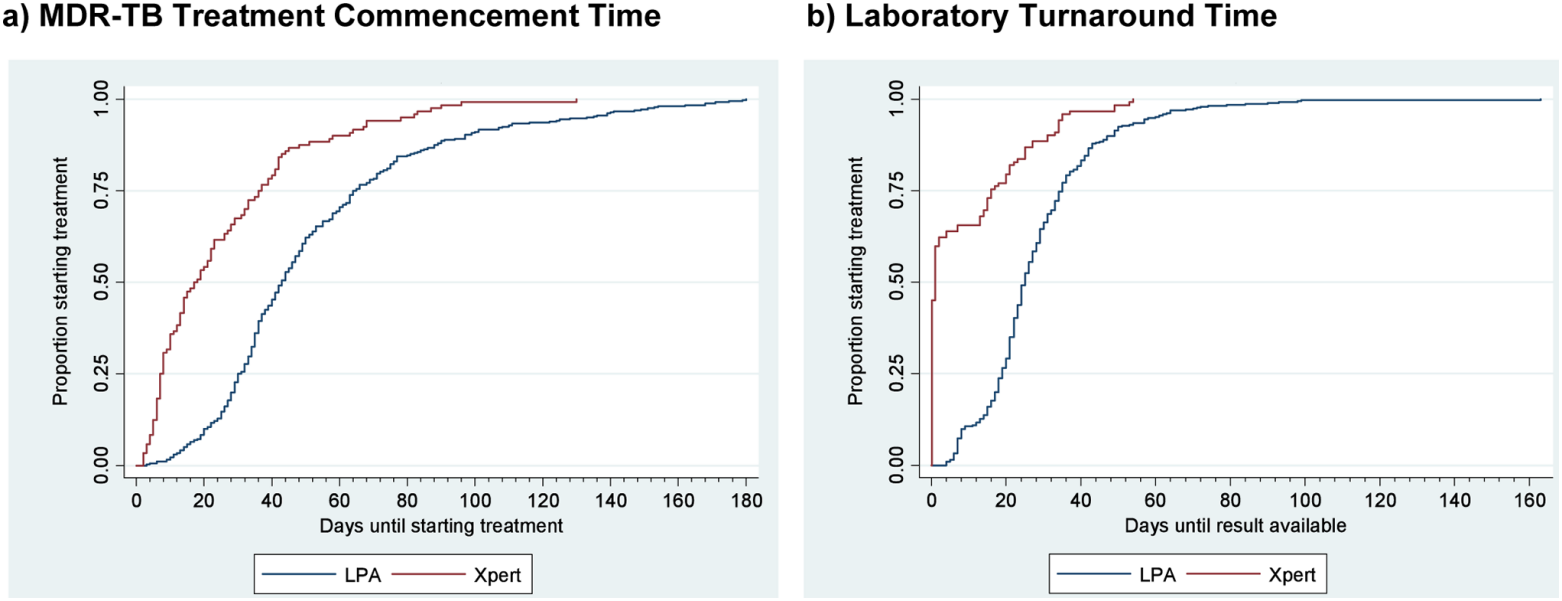
Cumulative Time-to-event Plots by Algorithm. Kaplan Meier time-to-event plots are shown for MDR-TB treatment commencement time (sample taken to treatment commencement) in Figure 3a and for laboratory turnaround time (to result available in the laboratory) in Figure 3b for cases included in the final analysis in the LPA- and Xpert-based algorithms. Abbreviation: MDR-TB - multidrug-resistant tuberculosis.

There was a reduction in median time-to-event between the LPA and Xpert-based algorithms (Table 2): MDR-TB TCT was reduced from 43 to 17 days (Figure 3a); laboratory turnaround time from 24 days to <1 day (Figure 3b) and ‘action’ delay from 14 to 10 days. The facility-level paired analysis showed a difference of 25 days (95% CI 17 to 32 days) (p<0.001) in median MDR-TB TCT, 20 days (95% CI 14 to 27 days) (P<0.001) in median laboratory turnaround time and 5 days (95% CI 1 to 9 days) (p = 0.015) in median ‘action’ delay between algorithms.

**Table 2:** MDR-TB TCT, Laboratory Turnaround Time and Action Delay by Algorithm.

		LPA-based Algorithm	Xpert-based Algorithm
MDR-TB TCT (days)	Median (95% CI)	43 (40–46)	17 (13–22)
	*Interquartile range*	*30–64*	*7–36*
Laboratory Turnaround Time (days)	Median (95% CI)	24 (22–25)	<1 (<1–1)
	*Interquartile range*	*18–33*	*<1–17*
Action delay (days)	Median (95% CI)	14 (13–15)	10 (8–14)
	*Interquartile range*	*9–30*	*6–21*
Median MDR-TB TCT fordifferent categories ofpatients (days) (95% CI)	Female	43 (37–47)	14 (10–19)
	Male	43 (40–47)	22 (14–29)
	HIV-positive	43 (40–47)	17 (12–28)
	HIV-negative	44 (36–49)	17 (8–22)
	Low MDR-TB risk	42 (38–46)	14 (10–27)
	High MDR-TB risk	44 (40–49)	18 (13–23)
	MDR-TB diagnostic time-point:Pre-treatment	43 (39–47)	14 (10–20)
	MDR-TB diagnostic time-point:Treatment	43 (38–48)	36 (19–51)
	MDR-TB treatment initiationTB hospital	44 (34–52)	23*
	MDR-TB treatmentinitiation PHC facility	43 (40–46)	16 (13–22)

Table showing median time-to-event for cases included in the final analysis in each algorithm. Abbreviations: MDR-TB TCT - Multidrug-resistant tuberculosis treatment commencement time’ HIV - human immunodeficiency virus; CI – Confidence interval; PHC – primary health care. *95% CI not reported due to small sample (n = 2).

**Figure 4:**
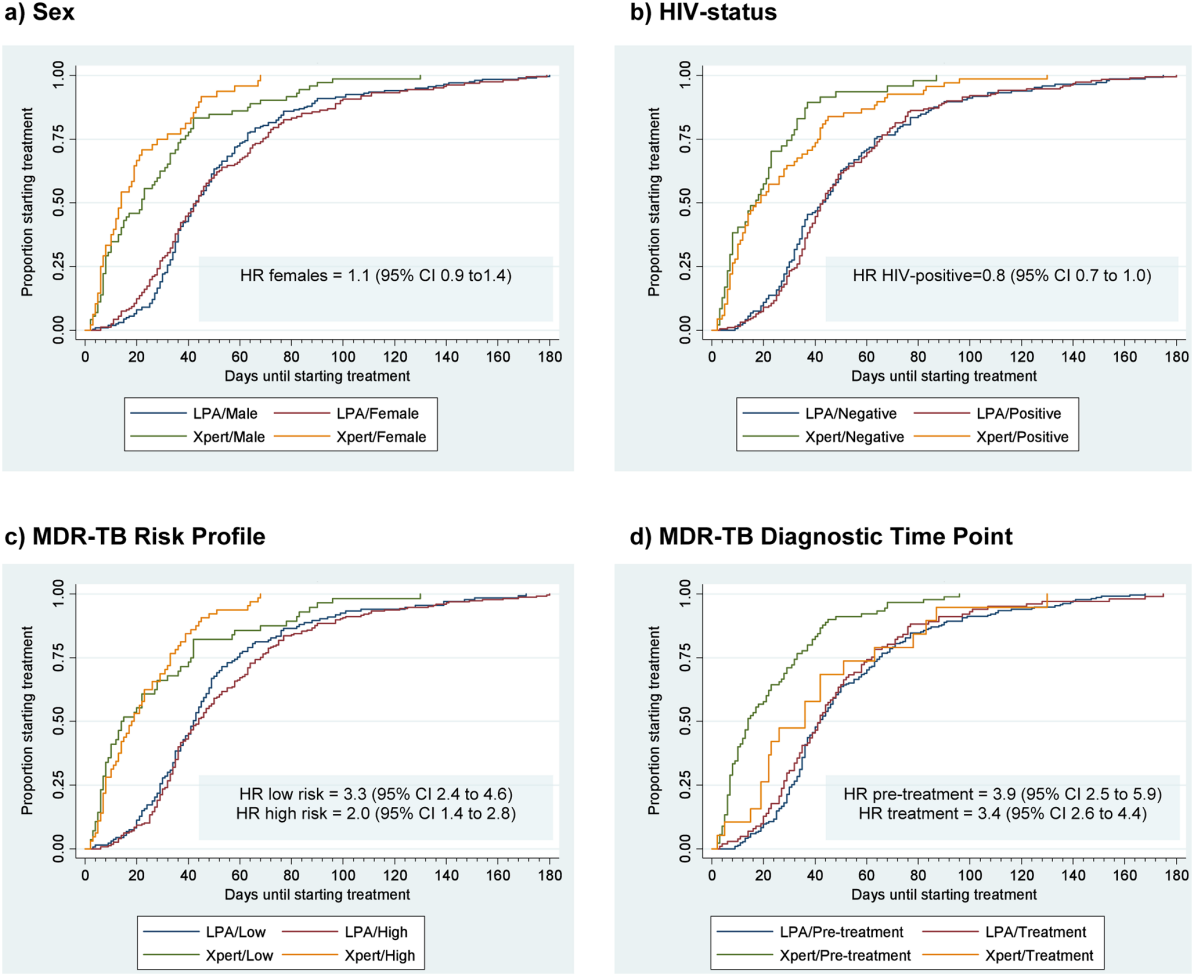
Cumulative Time to MDR-TB TCT Plots for Co-variables Assessed by Algorithm. Kaplan Meier time-to-event plots are shown for patient level variables assessed in the LPA-and Xpert-based algorithms: a) Sex; b) HIV-status; c) MDR-TB Risk Profile and d) MDR-TB Diagnostic Time-point. Inserts show Hazard Ratio (HR) for the univariate Cox regression analysis. Abbreviations: MDR-TB - multidrug-resistant tuberculosis; TCT – treatment commencement time.

A univariate analysis showed no significant association between MDR-TB TCT and age (p = 0.429), sex (p = 0.064) (Figure 4a), HIV-status (p = 0.056) (Figure 4b) or treatment initiation site (p = 0.340). There was a significant association between MDR-TB TCT and MDR-TB risk profile (p = 0.032): TCT decreased for both risk profiles (Figure 4c), but more so in the low-risk category (hazard ratio (HR) 3.3, 95% CI 2.4 to 4.6, p<0.001) than the high-risk category (HR 2.0, 95% CI 1.4–2.8, p<0.001). A significant association was also found between MDR-TB TCT and the MDR-TB diagnostic time-point (p = 0.001): the difference was greater for cases at the pre-treatment diagnostic time-point (HR 3.9, 95% CI 2.5 to 5.9, p<0.001) than at the treatment time-point (HR 3.4, 95% CI 2.6 to 4.4, p<0.001) (Figure 4d).

However, in the extended Cox regression model that adjusted for all these patient level variables, only the algorithm produced a significant effect, with a hazard ratio of 2.7 (95% CI 2.1 to 3.4, p<0.001) in the Xpert compared to LPA-based algorithm.

## Discussion

This is one of the first studies to report on MDR-TB TCT in an Xpert-based algorithm under routine operational conditions. A reduction of 25-days in median MDR-TB TCT was found with the introduction of the Xpert-based algorithm. Most of the gain (80%) resulted from a reduced laboratory turnaround time with only 20% due to a reduction in the ‘action’ delay.

In this before-and-after comparison, a range of health system improvements that were introduced may have contributed to the reduction in MDR-TB TCT in the Xpert-based algorithm. At PHC-level, for example, care was fully decentralised for patients not requiring hospitalisation. From 2012, standard MDR-TB drug regimens were made available at PHC-level and sub-district medical officers could initiate treatment without prior review of cases or prescriptions from the TB-hospital. A nurse was also employed in each of the eight sub-districts to trace MDR-TB patients, refer to appropriate social services, arrange screening of contacts and ensure work-up and treatment commencement.

Considering the median laboratory turnaround time of <1 day and the health system improvements that were introduced, the median MDR-TB TCT of 17 days (95% CI 13 to 22 days) in the Xpert-based algorithm showed an unexpected level of delay. This partly reflected the time taken for pre-MDR-TB treatment clinical requirements such as chest x-rays, liver function tests and audiometry (done centrally at the TB hospital). Since the Xpert algorithm was only introduced for a period of 18 months during the study, it is possible that as the changes that have been introduced are entrenched, further reductions in MDR-TB TCT will be achieved.

Several factors may have contributed to ‘action’ delays, including inefficiencies in accessing results and recalling patients. ‘Action’ delays have also been found in other studies. In the Western Cape of South Africa, Jacobsen et al [18] found median delays from result being sent to the facility to treatment commencement of 20 days with LPA compared to 19 days with conventional DST. In two health regions in Peru, Yagui et al [23] showed that delays due to slow bacterial growth on solid media and “action” delays at various time-points contributed equally to the median TCT of almost 5 months. Patient factors may have also contributed to delays [24], including patients’ failure to return promptly for their results due to work and family commitments and to perceptions of long waiting times and poor services.

There was no significant difference in MDR-TB treatment non-initiation rates between algorithms, due possibly to the small sample size in the Xpert-based algorithm. However, we found substantially lower non-initiation rates in both algorithms than those reported for South Africa [4], [25]. The 6-month cut-off used in our definition of non-initiation has contributed to this. Standardisation of the definition will enable comparisons between studies. It is unclear to what extent the changes in the health systems may have also contributed and this requires further investigation.

As was expected based on the health system changes, a higher percentage of patients initiated treatment at PHC-level in the Xpert-based than in the LPA-based algorithm (98% compared to 88%, p<0.001). An unexpected finding however in the LPA-based algorithm was the similarity in MDR-TB TCT for patients initiating treatment at the TB-hospital compared to at PHC-level with a median of 44 and 43 days respectively. The need for prior case reviews and prescriptions from the TB-hospital for those initiating treatment at PHC-level may account for this. It is not possible to make inferences about the impact of decentralised care in the Xpert-based algorithm due to the small number initiating treatment at the TB-hospital.

The extent to which vulnerable groups benefit from a new diagnostic test is an important aspect of impact assessment [21]. The failure to find a significant reduction in MDR-TB TCT for HIV-positive individuals in the Xpert-based algorithm is surprising. Based on the increased sensitivity of Xpert for smear-negative TB cases [12], we expected to find that a higher proportion of HIV-positive individuals would be diagnosed by Xpert and would not require lengthy culture and DST. Our finding could however be attributed to the small sample in the Xpert-based algorithm and is a limitation of the study. There were also no benefits in MDR-TB TCT by age or gender.

New molecular tests need to be evaluated within the context of a diagnostic algorithm [21] and this is a unique aspect of this study. We found that not all patients in the Xpert-based algorithm received an Xpert test: 17% of this group were TB cases evaluated through culture and LPA when a first-line regimen failed. Studies that report on TCT based solely on a positive Xpert test fail to take this and other factors into account, including cases in whom the correct test was not requested or could not be done (due to an inadequate sputum volume, for example).

Whilst an operational evaluation provides important insights into the benefits possible in real-world settings, it has limitations. The quality and completeness of routine data is the first of these. Clinical records did not provide adequate information to assess the time between the onset of symptoms and MDR-TB testing. The 25-day reduction in TCT needs to be viewed in relation to this delay.

Another limitation of the study is that MDR-TB TCT was calculated from the point at which the MDR-TB test was taken and not necessarily the starting point on the algorithm. Treatment delay was thus potentially underestimated in the LPA-based algorithm as new TB cases did not have initial DST and may have had undiagnosed primary MDR-TB when evaluated for TB. We also did not assess the impact of the algorithm on MDR-TB treatment outcomes.

The extent to which our results can be generalised is limited by the setting: all facilities in the study were urban or peri-urban; Cape Town has a relatively good laboratory and health infrastructure with access to rapid liquid culture and decentralised MDR-TB treatment. During the study period all tests were done at a central laboratory. Additional evidence is therefore required from studies in rural settings, where liquid culture is not available and where there is decentralised use of Xpert, to provide a broader understanding of potential benefits.

In South Africa, where Xpert has been introduced as a replacement for smear microscopy, annual TB diagnostic costs are estimated to increase by 53–57% to USD 48–70 million per year at full Xpert coverage [26]. The reduced MDR-TB TCT in the Xpert-based algorithm needs to be assessed within the context of the cost-effectiveness of the algorithm. A more thorough understanding of impact also requires an assessment of other potential benefits including, for example, TB yield, treatment outcomes and benefits from a patient’s perspective.

## Conclusion

We require evidence that new diagnostic tests which perform well in controlled settings can have an impact when implemented in operational settings [27]. This study showed that median MDR-TB TCT was reduced by 25 days with the introduction of an Xpert-based algorithm in a routine operational setting. However, when considered against a median laboratory turnaround time of <1-day, the median TCT of 17-days in the Xpert-based algorithm showed an unacceptable level of delay, exceeding the national target of five days [28].

Despite the substantial investment in the new technology [26], patients did not benefit fully from the use of Xpert, due possibly to both health system and patient factors. These need to be evaluated and addressed. Strengthening the health care system is important in controlling MDR-TB [29]; unless health system improvements are actively pursued, the full benefits of the rapid laboratory test are unlikely to be realised.
